# The ABC Guide to Fluorescent Toolsets for the Development of Future Biomaterials

**DOI:** 10.3389/fbioe.2019.00005

**Published:** 2019-01-23

**Authors:** Ferdinandus, Satoshi Arai

**Affiliations:** ^1^Waseda Bioscience Research Institute in Singapore, Singapore, Singapore; ^2^Research Institute for Science and Engineering, Waseda University, Tokyo, Japan; ^3^PRIME-AMED, Tokyo, Japan

**Keywords:** fluorescent probes, bioimaging, chemical indicators, fluorescent proteins, single-cell studies

## Abstract

In recent decades, diversified approaches using nanoparticles or nano-structured scaffolds have been applied to drug delivery and tissue engineering. Thanks to recent interdisciplinary studies, the materials developed have been intensively evaluated at animal level. Despite these efforts, less attention has been paid to what is really going on at the subcellular level during the interaction between a nanomaterial and a cell. As the proposed concept becomes more complex, the need for investigation of the dynamics of these materials at the cellular level becomes more prominent. For a deeper understanding of cellular events, fluorescent imaging techniques have been a powerful means whereby spatiotemporal information related to cellular events can be visualized as detectable fluorescent signals. To date, several excellent review papers have summarized the use of fluorescent imaging toolsets in cellular biology. However, applying these toolsets becomes a laborious process for those who are not familiar with imaging studies to engage with owing to the skills gap between them and cell biologists. This review aims to highlight the valuable essentials of fluorescent imaging as a tool for the development of effective biomaterials by introducing some cases including photothermal and photodynamic therapies. This distilled information will be a convenient short-cut for those who are keen to fabricate next generation biomaterials.

## How do Nanomaterials Affect Cellular Activities?

Recent advances in the development of biomaterials have yielded various types of nanomaterials for medical imaging, drug delivery, and regenerative medicine. The words nanomaterials and nanomedicine increasingly appear in these studies (Wagner et al., 2006). A nanometer-sized object is compatible with the scale of a single cell. For instance, drug-containing nanoparticles or nanorods are able to be taken up into a micron-sized cell efficiently, reach subcellular compartments, and lead to the alteration of cellular activities (Peer et al., 2007). To further benefit therapeutic efficiency, the surface of the nanomaterials can be modified to target the cells of interests (Chauhan and Jain, 2013; Yhee et al., 2014). Apart from nano-sized objects, two or three dimensional bulk-sized materials have also emerged, where their thickness is in the order of nanometers and/or their surface possesses a sophisticated nanostructure (Fujie, 2016). These nanomaterials effectively interact with biomacromolecules located at the cellular surface at a nanoscale interface (Luo et al., 2013). As potential applications, the modification of medical devices decorated with functional surfaces can prevent undesirable side effects, such as immune responses, when they are introduced into the body (Franz et al., 2011). Also, customized culture dishes with nanostructured surfaces enable the induction of cellular differentiation and the morphological control of multicellular components (Discher et al., 2005; Marino et al., 2015b).

When developing ideas for biomedical applications, one starts with the design of a nanomaterial that can alter cellular functions at the subcellular level. After a proof of concept is demonstrated in simple cellular studies, one may go further and carry out studies in animal models. In recent years, material scientists carried out animal studies themselves due to the growth in interdisciplinary studies between medicine and material science. However, despite these efforts, the evaluation of whether the designed material is really able to function as expected has been overlooked. In particular, little is known about the spatiotemporal information in real time regarding the behavior of the nanomaterials in cells.

**Figure 1:**
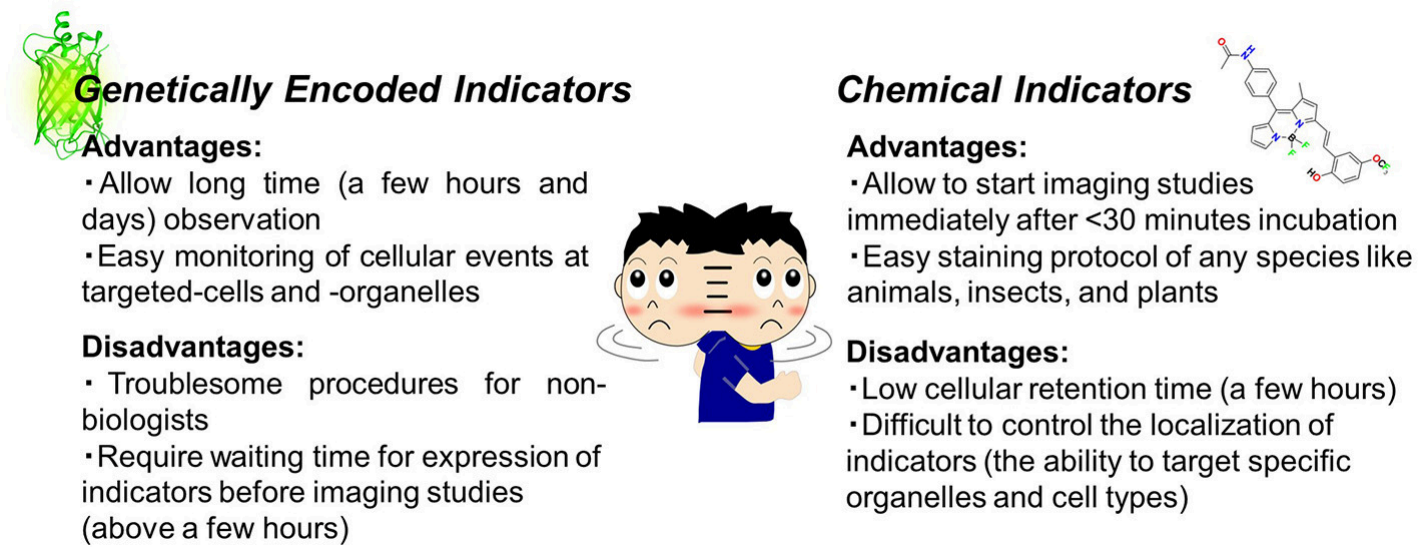
Chemical indicators vs. genetically encoded indicators (PDB ID: 3DPW).

Thanks to advances in molecular and chemical biology, various toolsets are easily accessible to light up cellular events using fluorescence imaging. As the first step, it is necessary to choose an adequate microscope (epi-microscope, confocal microscope, etc.) and then make a decision on the appropriate fluorescent indicators (probes or sensors). Although the development of indicators has progressed tremendously in chemistry and biology, the strategy adopted in both fields is likely to be common, which means that these indicators are capable of reporting changes in intracellular events as fluorescent signals. In general, the indicators are classified into small chemical or genetically encoded indicators. This review will skip over detailed descriptions as these have already been reviewed in depth (Giepmans et al., 2010; Kang et al., 2011; Newman et al., 2011; Specht et al., 2017). In brief, genetically encoded indicators can specifically target organelles or events and exhibit long-term (for example, days) stability under microscopic observation (Greenwald et al., 2018). On the other hand, chemical indicators are easy to handle and thus accessible for anyone without the need for expertise in cell biology (Figure 1). Owing to recent developments, there is a risk to oversimplify the difference between the two types of indicators. For instance, the modified Baculovirus allows a safe and easy-handling protocol for genetically encoded indicators for researchers without any molecular biology skills. Such technology could contribute to solve the gap between the two indicators in future.

This review paper stresses the significance of the interaction between nanomaterials and biospecimens at the submicron scale. It therefore focuses on the tools for observing the dynamics of cellular events in real-time rather than simple immune staining. We will also skip any references to commercially available kits or indicators.

## Toolsets to Visualize Intracellular Events in Real-Time

(i) How to See Changes in Cell State

Nano-sized carriers invade cells and then release drugs; so what happens to the cells and how do we visualize it? In the case of anticancer drug-loaded carriers, a cell viability assay (**live/dead assay kit**) could be the primary option. Of further interest would be the pathway through which cell death is executed, either apoptosis or necrosis. Necrosis involves the collapse of the outer cellular membrane, which can be evaluated with **propidium iodide (PI)**. During apoptosis, phosphatidylserine appears on the outer leaflet of the lipid bilayer, which can be identified with **fluorescent labeled Annexin V**. Looking at apoptosis more closely, indicators for mitochondrial membrane potentials (**JC1**) and Caspase activities (**Caspase-3/7 Green**) would be helpful.

To date, most drug delivery systems are designed to cause cell death in diseased cells and in such cases a simple live/dead test is frequently done. If the drug does not lead to complete cell death, it is possible to overlook the chance that nanomaterials could be affecting other cellular functions such as the cell cycle and proliferation. In fact, it was reported that even old-fashioned hyperthermia therapy perturbed the cell cycle (Dewey, 2009). In such cases, a genetically encoded indicator, Fluorescent Ubiquitination-based Cell Cycle Indicator (**Fucci**), could be used to distinguish S, G2, and M phases (Sakaue-Sawano et al., 2008).

In addition to the cell cycle, the change in energy metabolism resulting from nanomaterials is worth considering, though rarely discussed. A proliferating cell, such as a cancer cell, adopts a unique way to produce ATP as an energy source via strong glycolytic pathways, which is known as the Warburg effect (Heiden et al., 2009). Cancer cells therefore depend primarily on external glucose to survive while differentiated, non-cancerous cells rely on oxidative phospholyration (Oxphos) in mitochondria. ATP measurements are commonly performed using the well-known luciferin-luciferase assay (Manfredi et al., 2002). However, this method loses the spatiotemporal information of intracellular ATP dynamics. In order to visualize ATP fluctuations in real-time inside the cell, several fluorescent indicators have been developed, starting with genetically encoded indicators (**ATeam** and **Perceval**/**PercevalHR**), followed by the advent of small molecule indicators (Kurishita et al., 2012; Tantama et al., 2013; Tsuyama et al., 2013; Yaginuma et al., 2014). More recently, a team including an author of this review published a paper on the success of red, green, blue (RGB) color fluorescent indicators (**MaLions**) (Arai et al., 2018). This handy colorful toolset allows the observation of ATP dynamics at different organelles in the same cell simultaneously.

Other tools for visualizing energy metabolism include genetically encoded indicators sensing glucose (Ye and Schultz, 2003) and lactate (San Martín et al., 2013). These provide direct evidence for changes in metabolic pathways.

(ii) How to image Signaling molecules

In recent years, pioneering groups have attempted to generate stimulation devices that allow remote control of physiological functions in brains and muscles (Stanley et al., 2012; Marino et al., 2015a). The targeted functions are frequently associated with intracellular Ca^2+^ flux by external stimulation, which can be visualized using commercially available chemical indicators such as Fluo 4 and Fluo 8. Surprisingly, even a couple of decades after the advent of its prototype, Fluo 4 is still commonly used as a gold standard (Russell, 2011). Although there is no doubt regarding the viability of such indicators, their major drawback is their short cellular retention time. Due to the multiple carboxyl groups of the indicators in the anionic charged state, these chemical indicators are likely to be pumped out from cells via the anion transporter (Takei et al., 2013). On the other hand, genetically encoded Ca^2+^ indicators such as **GCaMP** enable long-term observations (Nakai et al., 2001). Moreover, the expanded color set of genetically encoded indicators prevails over chemical ones as color palettes, such as the R-, G-, B-**Geco** series, have already been established (Zhao et al., 2011). For more quantitative analysis, one should consider using the fluorescence resonance energy transfer (FRET)-based **Cameleon** series (Miyawaki et al., 1997). Considering its potential applications, the genetically encoded glutamate indicator, **iGluSnFr**, could be useful to visualize synaptic communications (Marvin et al., 2013). Also, as the other second messenger in addition to Ca^2+^, indicators to detect cAMP, such as the **Flamindo** series, are effective (Odaka et al., 2014; Harada et al., 2017).

Reactive oxygen species (ROS) play a critical role in several cascades. A commercially available indicator, 2′,7′-dichlorodihydrofluorescein diacetate (**H2DCFDA**), is capable of capturing any kind of ROS non-specifically. For advanced studies, more specific indicators are available for distinguishing between several ROS species (hydroxyl radical; **OxiOrange**^**TM**^, HClO; **HySOx**, H_2_O_2_; **HYDROP**, ONOO radical; **NiSPY-3**, Molecule Oxygen; **LOX-1**). Although limited to H_2_O_2_, genetically encoded indicators (**Hyper** series) are also applicable for monitoring ROS dynamics at target organelles (Bilan et al., 2013).

(iii) How to see invisible physicochemical elements

Many biomedical applications, such as therapeutic devices and nanomaterials, are associated with external physical stresses, such as force, magnetic fields, and temperature. For example, how does a single cell experience the temperature change produced by heat therapeutic devices? **ER thermo yellow** (or **ERthermAC**) and **Mito thermo yellow** are available to monitor temperature changes at the ER (endoplasmic reticulum) and mitochondria, respectively; these were developed by teams including an author of this review (Arai et al., 2014, 2015). As genetically encoded indicators, **gTEMP** and **tsGFPs**, which can target organelles, were developed (Kiyonaka et al., 2013; Nakano et al., 2017) and allow thermometry at the target organelles.

In addition to temperature as an obvious physical parameter for biomedical applications, there are several other elements to be examined in the biophysics field. For instance, several indicators were reported to visualize invisible factors, such as viscosity (Battisti et al., 2013; Liu et al., 2014), molecular crowding (Boersma et al., 2015), polarity (Sunahara et al., 2007; Abbandonato et al., 2018), and tension (Grashoff et al., 2010). Although the importance of these factors for biomaterials development still remains vague, these options should be kept in mind.

One may also have the interests in measuring the change of intracellular pH as a common physicochemical parameter. To monitor the pH, small chemical indicators (**SNARF** series) and genetic ones (**pHluorin** series) are available, some of which can be applied for two photon, ratiometric, and fluorescence life time imaging (Miesenböck et al., 1998; Bizzarri et al., 2009; Shen et al., 2014).

(iv) How to avoid the misinterpretation due to Artifacts

It is fascinating that a wide range of indicators are available for detecting various elements, however, one should take great care regarding pH fluctuations. Most indicators, regardless of whether they are genetically encoded or are chemical, follow the mechanism that the fluorescence intensity is altered as the change in the concentration of targeted analyte. Yet, the fluorescence of many indicators is also sensitive to pH changes owing to their pH-sensitive components, such as phenol and carboxylic groups. Though the cytoplasmic pH is maintained around 7.4 (different organelles being slightly different), the metabolic stress may cause an acidic change (Matsuyama et al., 2000). When the experimental conditions result in exposure to severe pH changes, this can be accounted for using pH indicators. This is vitally important to clarify whether the fluorescence change originates from the pH fluctuation or from the change in the concentration of targeted analyte (Berg et al., 2009). For example, one may correct the pH fluctuation using an additional pH indicator as reported (Berg et al., 2009). In another unique approach, Sato et al. published a success of simultaneous imaging of chloride ion and pH, where the pH correction was done using the indicator capable of detecting pH and chloride ion simultaneously (Sulis Sato et al., 2017). In addition to pH fluctuation, the fluorescence signal can also be affected by focus drift, different concentration of indicators, photobleaching, photoactivation, and several other variables. To avoid misinterpretation of the results, the detection limits of the indicators should be accounted for and the predicted artifacts considered.

## “Must have”, not “Nice to have”!

Although it is quite obvious that imaging technology is a powerful tool for the development of biomaterials, most researchers consider it as supplementary, that is, “nice to have,” but not “must have.” We would like to stress the importance of imaging studies as the concept of the materials gets more advanced. In this section, we present a few examples of the effective use of indicators.

(i) What happens inside a cell during “photothermal therapy”?

Thermal therapy to kill tumors by means of elevated temperature is a popular therapeutic approach as medical treatment for cancers, in which one of key things is the mechanism to heat up cells and tissues. Among several thermal therapeutic ways, photothermal therapy (PTT) has been the most appealing means which enables local treatment with minimal invasiveness. This method requires photothermal materials which absorb near infrared (NIR) light and convert the energy into heat. To date, innumerable materials such as inorganic materials, organic dyes, and semicondutive polymers, have been fabricated in order to achieve more efficient photothermal therapeutic effect (Zhang et al., 2013, 2015). However, these materials also possess the risks of generating ROS, aside from producing heat. In some cases, ROS is harmful for normal tissues and cells, thus making the development of a pure photothermal material challenging. Recently, Jung et al. successfully generated novel photothermal materials using the organic dyes, cryptocyanines, which are ineffective ROS generators and also specifically target the mitochondria (Jung et al., 2017). It is easy to imagine that a strategy to target a critical organelle would benefit therapeutic efficiency. The team clarified the mechanism using a fluorescent ROS indicator targeting mitochondria (**MitoSOX**); the heat generated from the dye by photoirradiation perturbed the electron transport chain, resulting in a change in endogenous ROS dynamics at the mitochondria, leading to cell death. In other words, the change in ROS dynamics is not triggered by ROS derived from the materials.

**Figure 2:**
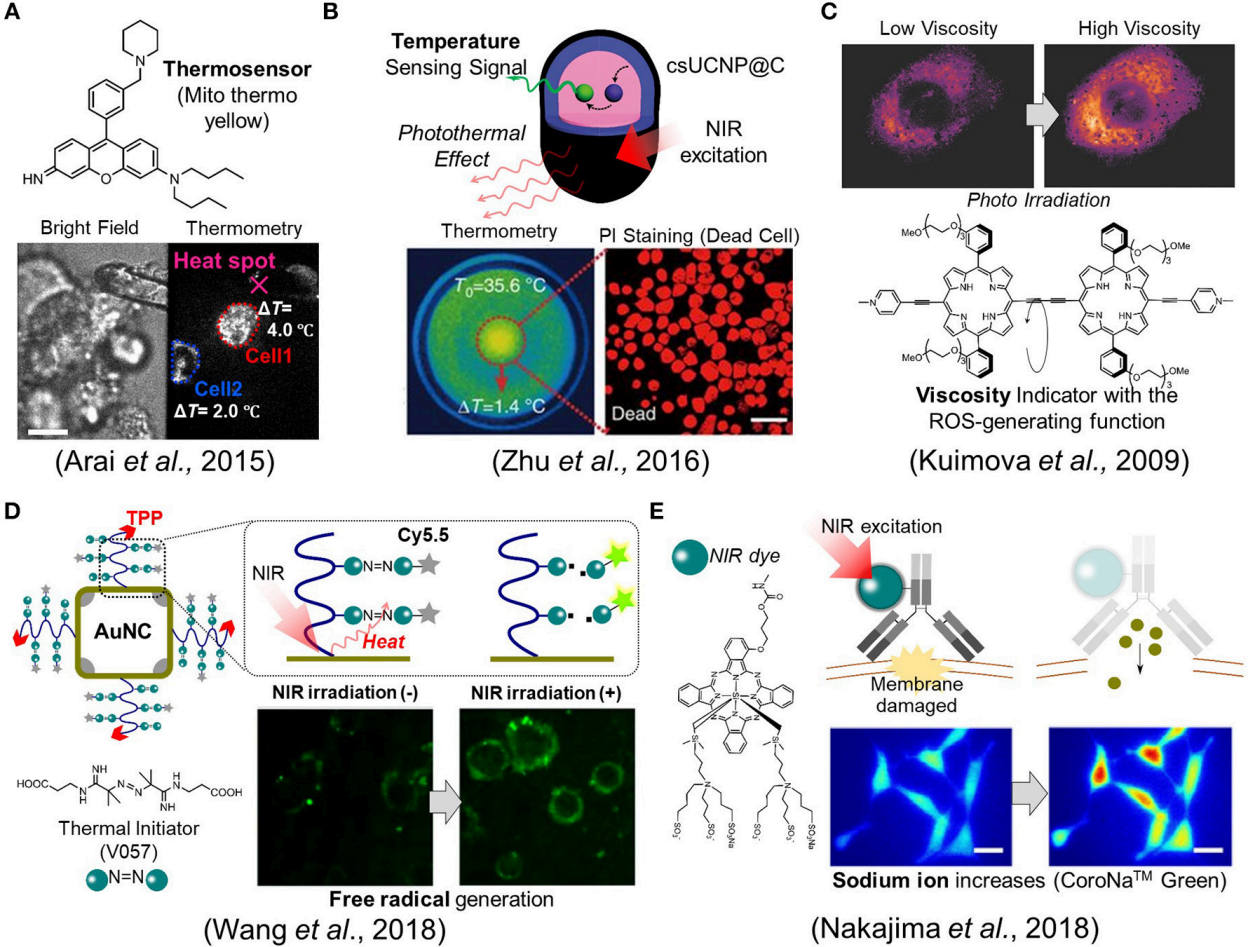
Fluorescence imaging studies in biomaterials development. **(A)** A mitochondrial targeting temperature indicator (Mito thermo yellow) detected the different temperature increment of each cell within a bunch of HeLa cells. Scale bar, 10 μm. **(B)** The nanocomposite, csUCNP@C, which comprises a phothermal heater and a thermometer, allows the induction of cell death while concurrently measures the temperature. Scale bar, 50 μm. **(C)** The porphyrin-dimer functions as a photosensitizer and an intracellular viscosity indicator. **(D)** The gold nanocage (AuNC) which acts as an efficient photothermal material was decorated with the thermal initiator (V057) and a fluorescent dye (Cy5.5). The free radical species was generated following the cleavage of V057 by photothermal effect. The free radical generation could be monitored with the fluorescence intensity change of Cy5.5. **(E)** The ion indicator, CoroNa^TM^ Green, detected the sodium ion influx during photoimmunotherapy. Scale bar, 20 μm. Reproduced with permission from **(A)** (Arai et al., 2015); copyright Royal Society of Chemistry, **(B)** (Zhu et al., 2016); copyright Nature Publishing Group, **(C)** (Kuimova et al., 2009); copyright Nature Publishing Group, **(D)** (Wang et al., 2018); copyright American Chemical Society, and **(E)** (Nakajima et al., 2018); copyright John Wiley & Sons.

As the other point in PTT, we need to pay attentions to how a single cell senses a rise in intracellular temperature caused by external heating. For example, when a temperature change was brought about in the multicellular spheroidal HeLa cells by external heating, a mitochondrial targeting temperature indicator, **Mito thermo yellow**, showed that each cell in the spheroidal aggregates experienced a different temperature change depending on its position (Arai et al., 2015) (Figure 2A). The maximum temperature difference between cells turned out to be around a couple of degrees even within the same bunch of cells. This result implies that maximal therapeutic efficiency cannot be achieved if heating is not homogeneous at the single cell level. This thermometry was also applied to evaluate the stimulation of skeletal muscles by heat (Marino et al., 2017). Attilio et al. demonstrated that once a gold nanoparticle, as a heat generator, was taken into the skeletal muscles, its contraction could be induced by heat with the irradiation of a NIR laser. A small fluorescent temperature indicator, ER thermo yellow, assisted in monitoring the intracellular temperature change while the muscle was contracting. For a more advanced example, Zhu et al. reported an intelligent nanocomposite that comprises a photothermal material and a fluorescent thermometer together (Zhu et al., 2016) which allows more accurate thermometry at the heat spot owing to the zero distance between the heater and the thermometer (Figure 2B).

(ii) What happens inside a cell during “photodynamic therapy”?

Much like photothermal therapy (PTT), photodynamic therapy (PDT) has a long history. However, there are scarce reports where cellular events were observed during the photodynamic therapy. For example, Kuimova et al. reported a unique porphyrin-dimer that possesses dual functionalities as an intracellular viscosity indicator as well as a photosensitizer to generate singlet oxygen species acting as cytotoxic agents (Kuimova et al., 2009) (Figure 2C). After photoirradiation, the generated singlet oxygen led to cell death while also causing a change in viscosity, which was visualized using their ratiometric viscosity indicator. Interestingly, it turned out that the increase in intracellular viscosity was not homogeneous and decreased the diffusivity of intracellular species. They suggested that the increase in the viscosity resulting from the photosensitizer altered the formation of singlet oxygen species and also their decay. The viscosity at subcellular compartmentalized spaces could be an important factor as it may also alter the transport efficiency of drugs.

Very recently, Wang et al. proposed a new type of therapeutic mechanism where the free radical generator is coupled with the NIR light (Figure 2D) (Wang et al., 2018). Unlike common previous approaches, their designed material does not generate ROS from intracellular oxygens but instead produces free radical species from the thermal initiator (V057: 2,2′-Azobis [N-(2-carboxyethyl)-2-methylpro-pionamidine] hydrate). More specifically, the gold nanocage (AuNc) which acts as an efficient photothermal material was decorated with V057 and a fluorescent dye (Cy5.5). Once the temperature is elevated on the surface of AuNc by NIR irradiation, the free radical is produced due to the cleavage of the thermal initiator, V057. Simultaneously, the free radical generation could be monitored with the fluorescence intensity change of Cy5.5 since the fluorescence is recovered from quenched state involved in the free radical production. Importantly, the material is also designed to target to mitochondria in order to maximize the therapeutic efficiency. Thus, the total system allows the induction of the cell death with concurrent monitoring of the free radical generation in the targeted mitochondria. It was also noted that this system works in both normoxic and hypoxic conditions because of the oxygen-independent principle.

(iii) Tackles for “unknown mechanism” and future perspective

Though not quite often, we sometime face complicated situations where a material works well in animals but its mechanism remains vague. In such cases, imaging technology can provide vital clues by visualizing the dynamics at a cellular level. Mitsunaga et al. pioneers in photoimmunotherapy, developed a near-infrared dye-labeled antibody that binds specifically to cancer cells and then effectively induces cell death via NIR illumination (Mitsunaga et al., 2011). The system is also able to work efficiently in animal level, although the mechanism still remains unclear at cellular level. Crucially, it is unlikely to result in ROS or heat production. Recently, Nakajima et al. investigated the mechanism regarding this photoimmunotherapy and then verified that the plasma membrane was damaged for a short time by observing the sodium ion influx using a fluorescent indicator (Nakajima et al., 2018). This minute damage on the membrane resulted in the increase (Figure 2E) of its permeability, cell swelling, and cell death. In this case, though it is not still perfectly understood, the imaging technique provided a hint to elucidate the unknown mechanism.

The importance of the physical stimulus will evolve following the development of biomaterials. This is because attempts to manipulate cellular functions using physical stresses is one of the biggest topics in biophysics, known as mechanobiology, providing fresh insights for material science. Pioneers in this field are dedicated to revealing how cells sense mechanical stress and what the molecular players are (Uhler and Shivashankar, 2017). Concurrently, leading biologists have also produced various tools to help unveil the sensing mechanism at a molecular level; these tools will be of benefit to material scientists as well in the development of novel biomaterials. Ideally, the development of indicators, that are applicable for two-photon and NIR imaging, should be also significant to achieve the subcellular resolution for *in vivo* imaging using animals (Hong et al., 2017). We positively believe the promising future of imaging studies which will claim their place as a “must have” approach in biomaterials development.

## Author Contributions

F and SA conceived and revised the manuscript.

### Conflict of Interest Statement

The authors declare that the research was conducted in the absence of any commercial or financial relationships that could be construed as a potential conflict of interest.
